# Supplementary material for: Bacterial transcriptional repressor NrdR – a flexible multifactorial nucleotide sensor
Source: FEBS J. 2025 Mar 3;292(12):3091–112. doi: 10.1111/febs.70037 (PMC12176259; doi:10.1111/febs.70037)
Supplement: Supplementary file 1 — Fig. S1. Binding of E. coli NrdR simultaneously loaded with dATP and either ATP or ADP to E. coli RNR promoters. Fig. S2. Nucleotides eluted with NrdR, analysed using HPLC. Fig. S3. Binding of E. coli NrdR loaded with different single adenosine nucleoside phosphates or without any effector (lowest row) to NrdR boxes. Fig. S4. Binding of E. coli NrdR loaded with all possible combinations of adenosine nucleoside phosphates to NrdR boxes in nrdAB, nrdHIEF and nrdDG promoters, determined by MST. Fig. S5. Binding of E. coli NrdR loaded with c‐di‐AMP, a combination of c‐di‐AMP and ADP and cAMP to NrdR boxes in nrdAB, nrdHIEF and nrdDG promoters, determined by MST. Fig. S6. Size exclusion chromatography of NrdR with different effectors at varying protein quantities. Fig. S7. Superposition of all Zn‐ribbon domain pairs from the crystal structures of the AMPPNP‐dATP‐bound and ADP‐dATP‐bound forms of EcoNrdR. Fig. S8. The angles between ATP‐cone and Zn‐ribbon domains form clusters. Fig. S9. Binding of nucleotides in the SeMet‐EcoNrdR‐dATP‐ATP complex. Fig. S10. Crystal packing in the ADP/dATP‐bound form of EcoNrdR. Fig. S11. Nucleotides in the ADP‐dATP structure. Fig. S12. ITC analyses of ligand binding to E. coli NrdR at 20 °C, 25 °C and 30 °C. Fig. S13. Local resolution maps for the cryo‐EM structures. Fig. S14. Cryo‐EM data processing workflow for the structure of the EcNrdR‐ATP‐dATP‐DNA complex. Fig. S15. Data acquisition parameters and data processing workflow for the ATP‐bound NrdR filament cryo‐EM dataset. Fig. S16. Retained nucleotides in E. coli NrdR (35 μm) after addition of effectors and desalting. Table S1. Promoter‐binding constants for effector nucleotide‐loaded E. coli NrdR. Table S2. Size exclusion chromatography of NrdR with different effectors at varying protein quantities: summary of eluted NrdR complexes. Table S3. Angles (in degrees) between ATP‐cone and Zn‐ribbon domains in the crystal‐ and cryo‐EM structures of EcoNrdR reported in this work, as well as [file FEBS-292-3091-s006.docx]

# **Supporting material**

**Bacterial repressor NrdR – a flexible multifactorial nucleotide sensor**

Inna Rozman Grinberg^1#^, Ornella Bimaï^1#^, Saher Shahid^1^, Lena Philipp^1^, Markel Martínez-Carranza^1^, Ipsita Banerjee^2¶^, Daniel Lundin^1^, Pål Stenmark^1^, Britt-Marie Sjöberg^1^, Derek T. Logan^2,3,4^*

^1^Department of Biochemistry and Biophysics, Stockholm University, SE-10691 Stockholm, Sweden

^2^Biochemistry & Structural Biology, Centre for Molecular Protein Science, Department of Chemistry, Lund University, Box 118, SE-22100 Lund, Sweden

^3^Lund Institute for Neutron and X-ray Science, Lund University, SE-221 00 Lund, Sweden

^4^SciLifeLab Lund, Cryo-EM for Life Science, Lund University, SE-221 00 Lund, Sweden

^#^shared first authors

^¶^deceased

*corresponding author


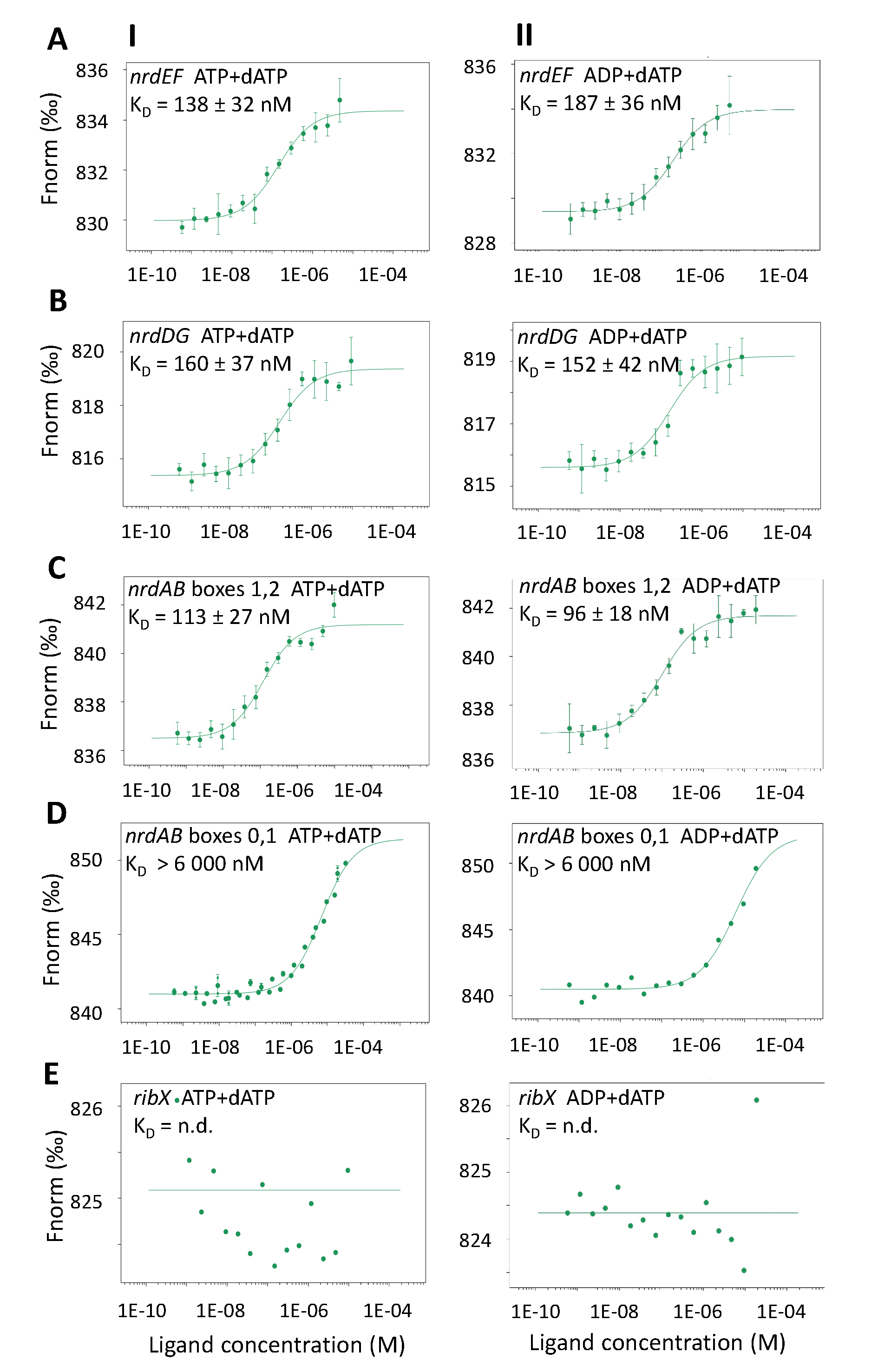


**Supporting Figure S1**. **Binding of *E. coli* NrdR simultaneously loaded with dATP and either ATP or ADP to *E. coli* RNR promoters *nrdHIEF* (A), *nrdDG* (B)*, nrdAB boxes 1, 2* (C) and alternative *nrdAB boxes 0, 1* (D) and *E. coli ribX* promoter (E) determined by MST.** Plots of the normalized fluorescence Fnorm (‰) from T-Jump and Thermophoresis vs. the concentration of ligand (NrdR) are shown. Lines represent fits of the data points using the K_D_ fit derived from the law of mass action. STDEV derived from at least three experimental repeats. In the case of nrdAB promoter oligo including NrdR boxes 0 and 1 the fits resulted in K_D_s in the range of 6 μM, but the actual K_D_ cannot be determined, since the curves do not reach a plateau.

**Supporting Table S1. Promoter binding constants for effector nucleotide loaded *E. coli* NrdR.**

| **Effector** | ***nrdHIEF* promoter**  K_D_ [nM] | ***nrdDG* promoter**  K_D_ [nM] | ***nrdAB* promoter box 1+2**  K_D_ [nM] | ***nrdAB* promoter box 0+1**  K_D_ [nM] |
| --- | --- | --- | --- | --- |
| ATP + dATP | 138 ± 32 | 160 ± 37 | 113 ± 27 | >6000 |
| ADP + dATP | 187 ± 36 | 152 ± 42 | 96 ± 18 | > 6000 |
| AMP + dATP | > 5 000 | > 5 000 | > 10 000 | > 10 000 |
| ATP + dADP | n.d. | n.d. | - | n.d. |
| ADP + dADP | 328 | 171 | 128 | > 3600 |
| AMP + dADP | n.d. | n.d. | - | n.d. |
| ATP + dAMP | n.d. | n.d. | - | n.d. |
| ADP + dAMP | n.d. | n.d. | - | n.d. |
| AMP + dAMP | n.d. | n.d. | - | n.d. |
| ATP | n.d. | n.d. | n.d | n.d. |
| ADP | > 100 000 | n.d. | > 5 000 | n.d. |
| AMP | n.d. | n.d. | - | n.d. |
| dATP | > 20 000 | > 6 000 | - | n.d. |
| dADP | n.d. | n.d. | - | n.d. |
| dAMP | n.d. | n.d. | - | n.d. |
| no effector | n.d. | n.d. | n.d | n.d. |
| cAMP | n.d. | - | - | - |
| Cyclic di-AMP | n.d. | n.d. | - | n.d. |
| Cyclic di-AMP + ADP | n.d. | > 100 000 | - | n.d. |

n.d.; not detected; -; not tested


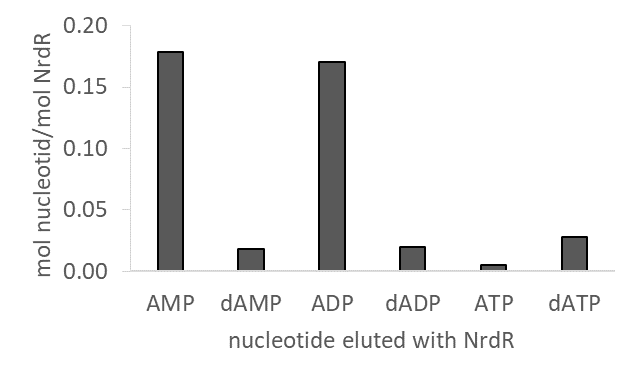


**Supporting Figure S2. Nucleotides eluted with NrdR, analysed using HPLC.** Recombinant *E. coli* NrdR (9 mg/ml, 495 µM) after nickel affinity purification and desalting was boiled for 10 min, centrifuged for 10 min at 17000 g on a table-top centrifuge, and loaded on HPLC (Agilent) using an Agilent ZORBAX RR StableBond (C18, 4.6 x 150 mm, 3.5 µm pore size) equilibrated with buffer A (10% methanol, 50 mM potassium phosphate buffer, pH 7, 10 mM tetrabutylammonium hydroxide). Sample of 10 μl was injected and eluted at 1 ml/min with a gradient of 40%-100% buffer B (30% methanol, 50 mM potassium phosphate buffer, pH 7, 10 mM tetrabutylammonium hydroxide). Compound identification and product quantification based on peak area were performed by external calibration using injected ATP, dATP, ADP, dADP, AMP and dAMP standards.


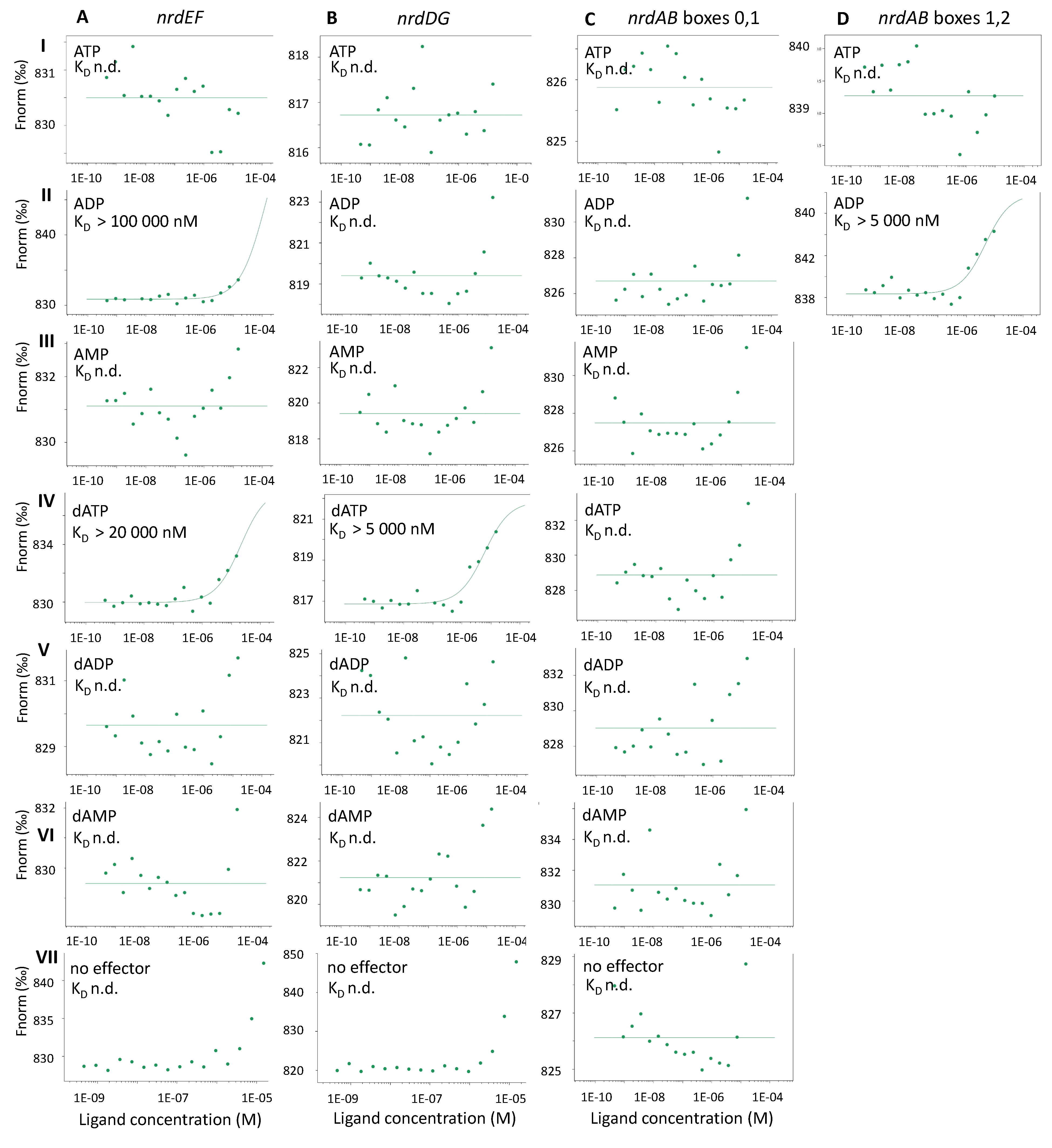


**Supporting Figure S3. Binding of *E. coli* NrdR loaded with different single adenosine nucleoside phosphates or without any effector (lowest row) to NrdR boxes in *nrdAB*, *nrdHIEF* and *nrdDG* promoters, determined by MST.** Plots of the normalised fluorescence Fnorm (‰) from T-Jump and Thermophoresis vs. the concentration of ligand (NrdR) are shown. Lines represent fits of the data points using the K_D_ fit derived from the law of mass action. Flat lines were produced in the cases where no fit could be generated by the software and the parameters therefore were fixed as for non-binder ligands. In most cases no binding was detected, and no fit could be generated and in individual cases the fits resulted in K_D_s in the range of 5 - 100 μM. The actual K_D_ cannot be determined, since the curves do not reach a plateau. Since these fitted K_D_s reflect binding affinities lower compared to those of dATP + ATP and dATP + ADP loaded NrdR (see table 1 in the main text), we believe that they are not physiologically relevant and reflect non-specific binding. n.d. not determined.


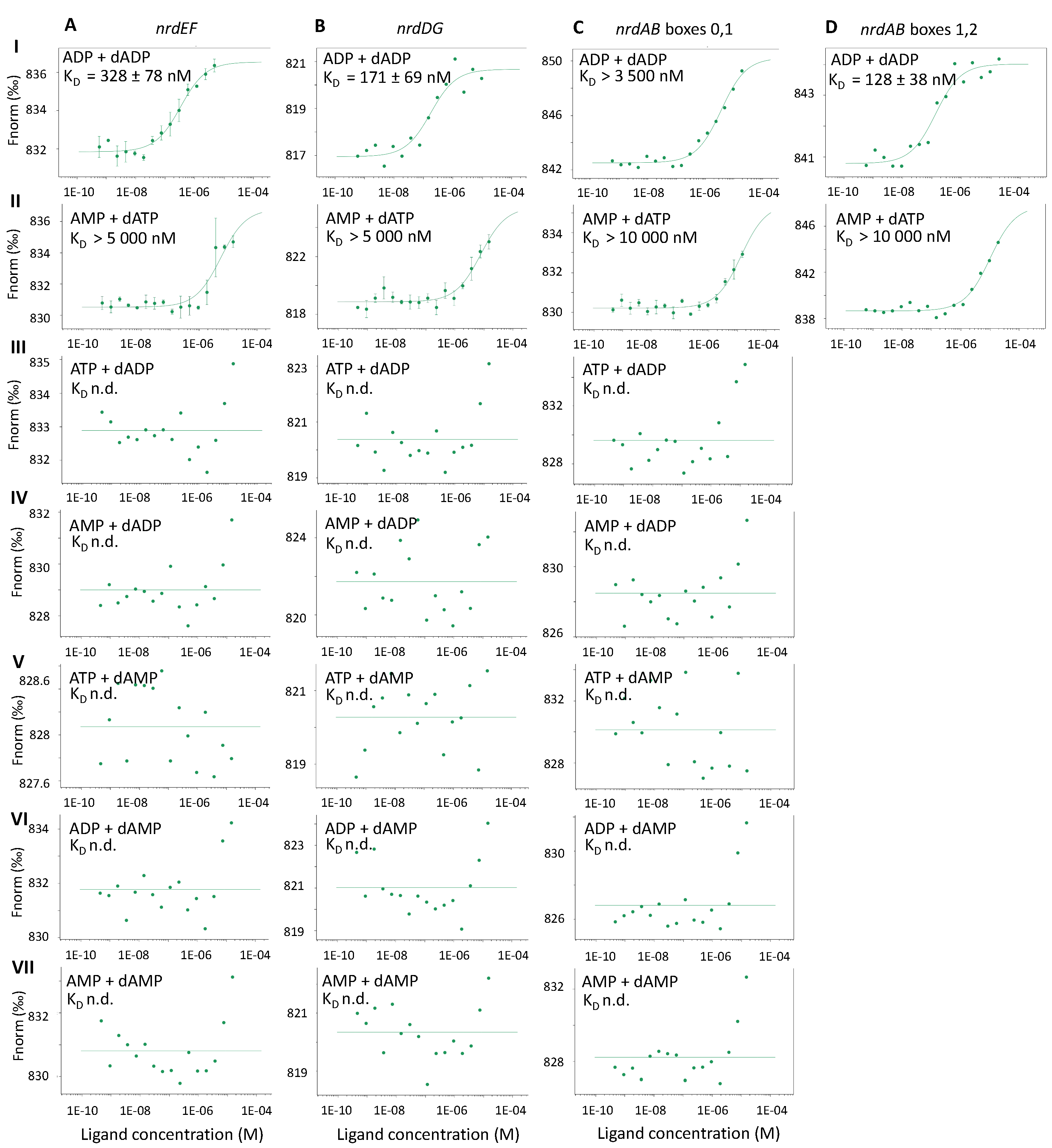


**Supporting Figure S4. Binding of *E. coli* NrdR loaded with all possible combinations of adenosine nucleoside phosphates to NrdR boxes in *nrdAB*, *nrdHIEF* and *nrdDG* promoters, determined by MST.** Plots of the normalised fluorescence Fnorm (‰) from T-Jump and Thermophoresis vs. the concentration of ligand (NrdR) are shown. Lines represent fits of the data points using the K_D_ fit derived from the law of mass action. Flat lines were produced in the cases where no fit could be generated by the software and the parameters therefore were fixed as for non-binder ligands. In some cases, the fits resulted in K_D_s in the range of 3.5 - 10 μM, but the actual K_D_ cannot be determined, since the curves do not reach a plateau. Since these fitted K_D_s reflect binding affinities lower compared to those of dATP + ATP and dATP + ADP loaded NrdR (see table 1 in the main text), we believe that they are not physiologically relevant and reflect non-specific binding. In many cases no binding was detected, and K_D_ couldn’t be fitted and therefore they are marked n.d. not determined.


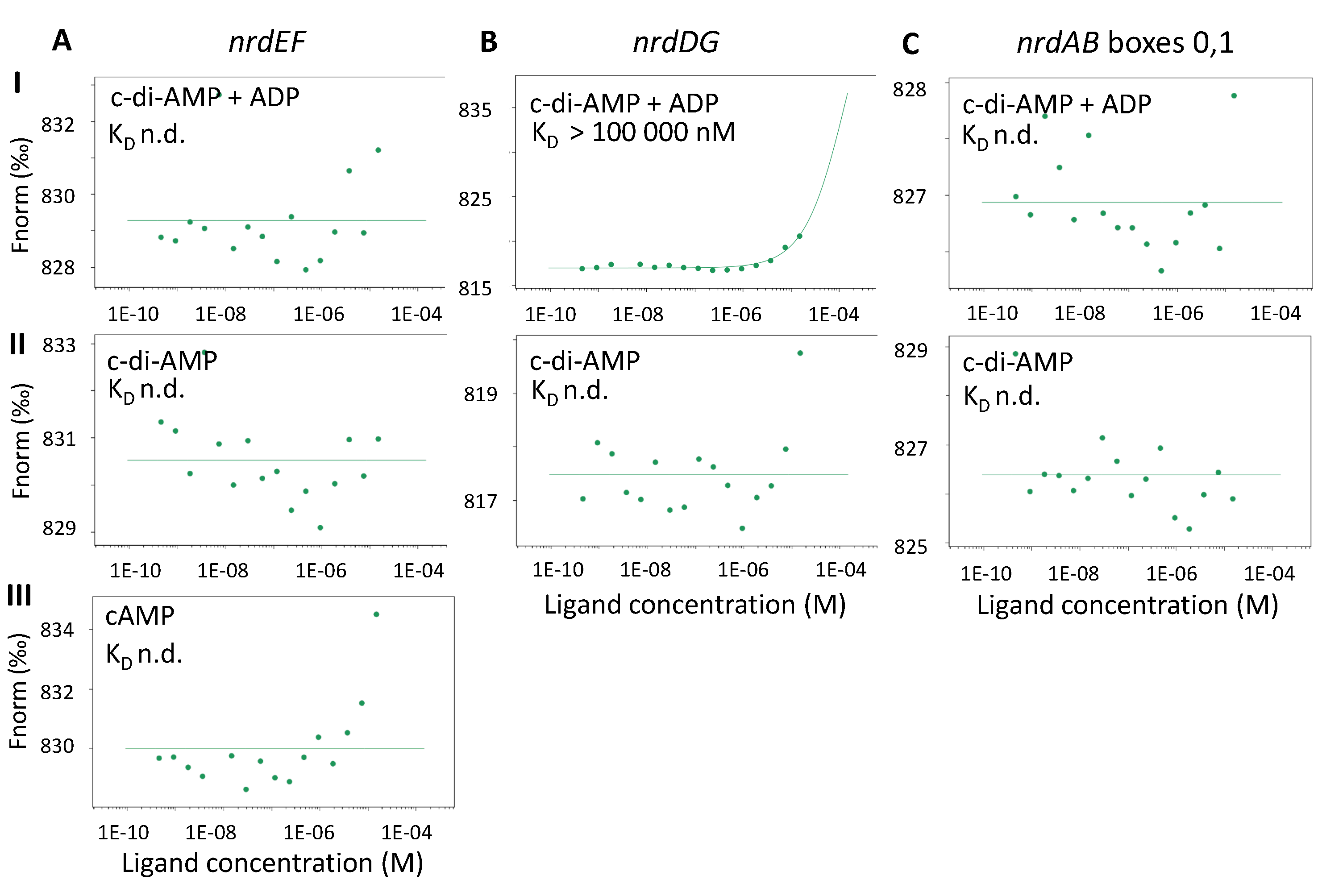


**Supporting Figure S5. Binding of *E. coli* NrdR loaded with c-di-AMP, a combination of c-di-AMP and ADP and cAMP to NrdR boxes in *nrdAB*, *nrdHIEF* and *nrdDG* promoters, determined by MST.** Plots of the normalised fluorescence Fnorm (‰) from T-Jump and Thermophoresis vs. the concentration of ligand (NrdR) are shown. Lines represent fits of the data points using the K_D_ fit derived from the law of mass action. Flat lines were produced in the cases where no fit could be generated by the software and the parameters therefore were fixed as for non-binder ligands. In the case of c-di-AMP- and ADP-loaded NrdR and the *nrdDG* promoter, the fit resulted in K_D_s higher than 100 μM and most likely reflecting non-specific binding. The actual K_D_ cannot be determined, since the curve doesn’t reach a plateau. Binding of NrdR loaded with cyclic AMP to DNA was assayed only with *nrdHIEF* promoter region. n.d. not determined.


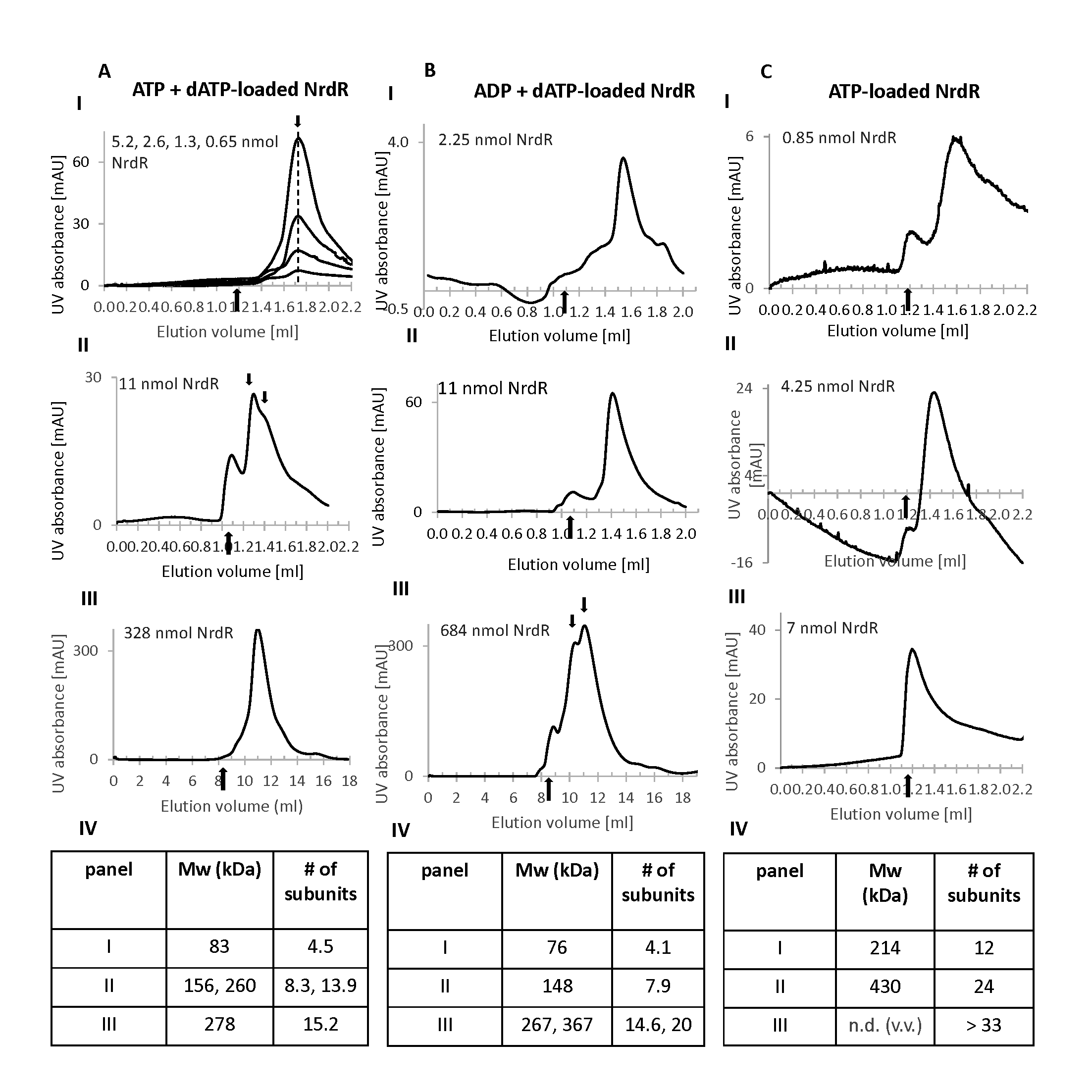


**Supporting Figure S6.** **Size exclusion chromatography of NrdR with different effectors at varying protein quantities.** Representative chromatograms are shown. **A.** NrdR supplemented with ATP and dATP. **AI**: 95 µl of 55, 27, 14 µM NrdR samples were loaded on a Superdex 200 PC 3.2/30 column connected to Shimadzu HPLC system. **AII**: 25 µl of 440 µM NrdR were loaded onto a Superdex 200 PC 3.2/30 column connected to an ÄKTA prime system. **AIII**: 0.6 ml of 547 µM NrdR was loaded onto a Superdex 200 gl 10/300 increase column (for purification prior to crystallisation) **B:** NrdR supplemented with ADP and dATP. **BI, BII**: 25 µl of 90 and 440 µM NrdR respectively were loaded on Superdex 200 PC 3.2/30 column connected to an ÄKTA prime system. **BIII:** 1 ml of 684 µM NrdR was loaded onto a Superdex 200 gl 10/300 Increase column (for purification prior to crystallisation) **C:** NrdR supplemented with ATP. **CI, CII, CIII:** 50 µl of 17, 85 and 140 µM NrdR samples were loaded on a Superdex 200 PC 3.2/30 column connected to a Shimadzu HPLC system equilibrated with buffer containing low MgCl_2_ and ATP. **A-C:** All NrdR samples were incubated with respective effector nucleotides before SEC and nucleotides were included in SEC buffer in all cases. See Materials and methods for further SEC details. Arrows indicate the peaks for which molecular weight was calculated. The column volumes of Superdex 200 PC 3.2/30 and Superdex 200 gl 10/300 Increase are 2.4 and 24 ml respectively. The column void volumes are marked with arrows on the x-axes. The apparent void volume of Superdex 200 PC 3.2/30 differs by ~0.1 ml between runs performed on the Shimadzu HPLC and the ÄKTA Purifier systems due to slightly different connecting tube volumes. The baseline noise is a consequence of gradual oxidation of DTT in SEC buffer and is noticeable in runs where low NrdR concentrations were used, as NrdR has low UV absorbance. A summary of eluted NrdR complexes is shown in Supporting Table S2.

**Supporting Table S2.** **Size exclusion chromatography of NrdR with different effectors at varying protein quantities: summary of eluted NrdR complexes.** Molecular weight of eluted NrdR complexes calculated based on standards for each column. Representative chromatograms are shown in Fig. S6. See legend to Fig. S6 and Materials and methods for details. The number of subunits was calculated based on molecular weight of NrdR of 18 280 Da.

| **Protein sample used,**  **panel in Fig. S6** | **nmol EcoNrdR loaded** | **Mw (kDa)** | **# of subunits** |
| --- | --- | --- | --- |
| **ATP-dATP-NrdR** |  |  |  |
| AI | 5.2, 2.6, 1.3, 0.65 | 83 | 4.5 |
| AII | 11 | 156, 260 | 8.3, 13.9 |
| AIII | 328 | 278 | 15.2 |
| **ADP-dATP-NrdR** |  |  |  |
| BI | 2.25 | 76 | 4.1 |
| BII | 11 | 148 | 7.9 |
| BIII | 684 | 267, 367 | 14.6, 20 |
| **ATP-NrdR** |  |  |  |
| CI | 0.85 | 214 | 12 |
| CII | 4.25 | 430 | 24 |
| CIII | 7 | n.d. (v.v.) | >33 |


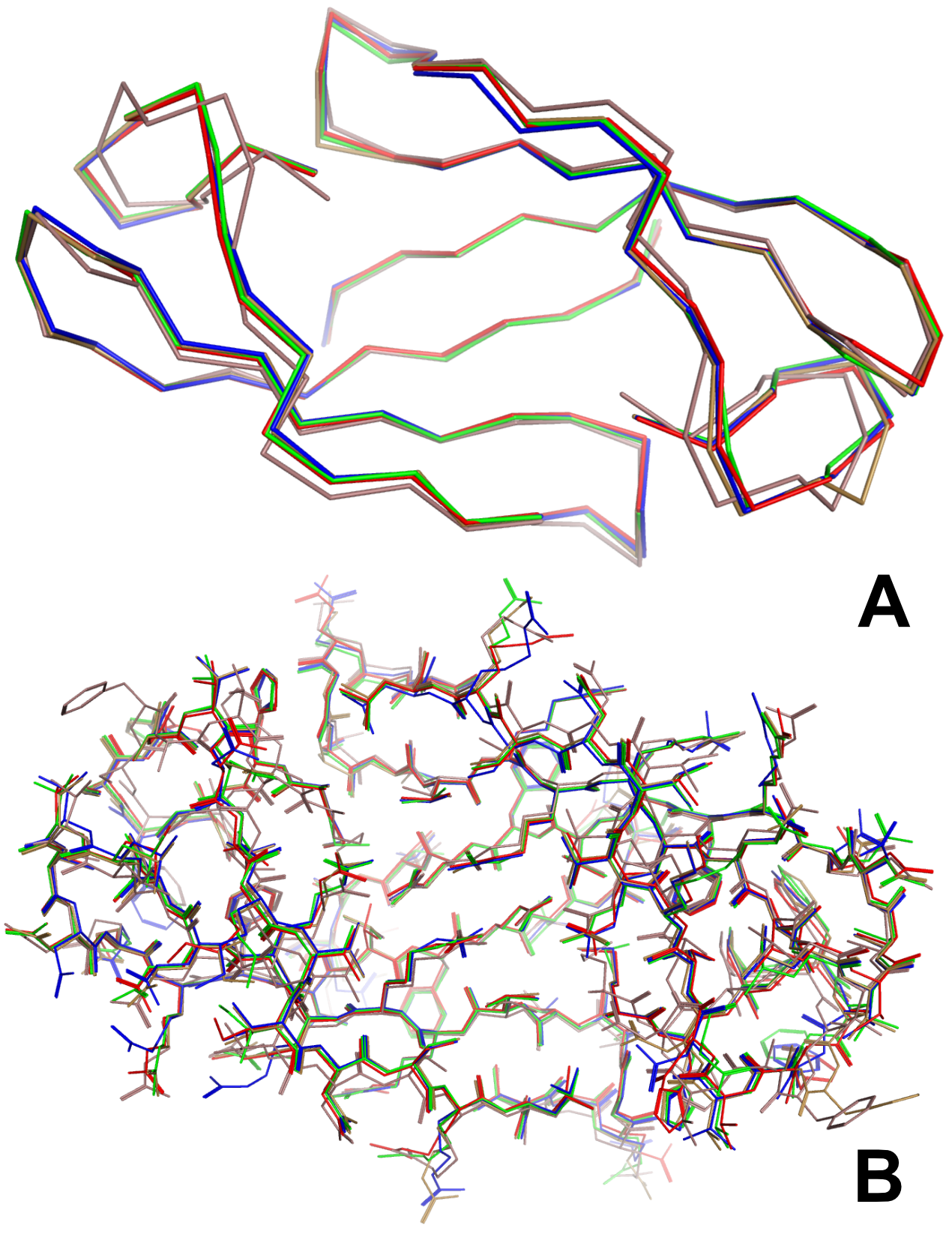


**Supporting Figure S7. Superposition of all Zn-ribbon domain pairs from the crystal structures of the AMPPNP-dATP-bound and ADP-dATP-bound forms of EcoNrdR**. A) backbone trace; B) all atoms. The four pairs from the AMPPNP-dATP-bound form are coloured red, green, blue and beige respectively, while the single pair from the ADP-dATP-bound form is coloured light purple. The deviations between the structures are almost exclusively in the side chains on the surface. A small deviation between the ADP-dATP form and the other pairs is seen in a loop on the top left / bottom right of the dimer, but this is most likely due to crystal packing effects. The structures support the observation that the Zn-ribbon pairs behave as rigid bodies.

**
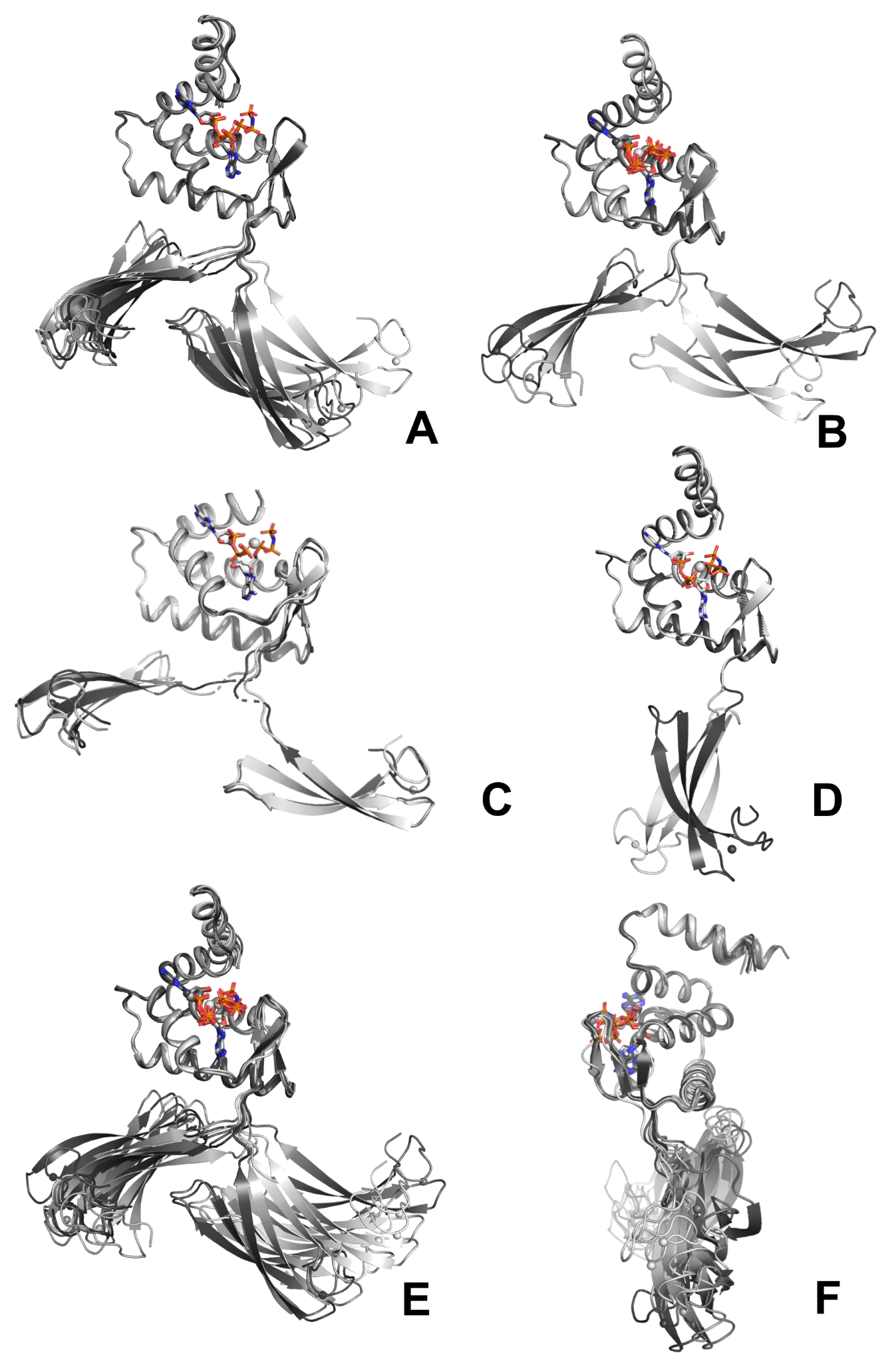
**

**Supporting Figure S8. The angles between ATP-cone and Zn-ribbon domains form clusters.** A) The eight polypeptide chains in the two independent tetramers of EcoNrdR-AMPPNP-dATP in the asymmetric unit, superimposed on the ATP-cone domains; B) The four chains of the single tetramer in the asymmetric unit of SeMet-EcoNrdR-ATP-dATP; C) Two chains from the EcoNrdR-ATP filament; D; The two independent chains of the EcoNrdR-ATP-dATP-DNA complex (refined with C2 symmetry); E) and F) Two orthogonal views of all 14 chains of structures except the DNA complex. This is in effect a superposition of panels A, B and C. The nucleotides in all structures are shown as sticks.

**Supporting Table S3. Angles (in degrees) between ATP-cone and Zn-ribbon domains in the crystal- and cryo-EM structures of EcoNrdR reported in this work, as well as the previously published structures of ScoNrdR.** The angle reported is the one subtended by the CA atoms of residues 81 and 98 (which define the ends of the helix in the ATP-cone closest to the Zn-ribbon domain) and the Zn atom of the Zn-ribbon (Figure 4C). For ScoNrdR the equivalent helix begins and ends at residues 78 and 95. Average values and standard deviations for the clusters formed by chains of type A and type B are given in the last four columns. For the ATP filament, only the angles for the central two tetramers are given, as the fit to the map is best in that region.

|  | **A** | **B** | **C** | **D** | **E** | **F** | **G** | **H** | **average cluster A** | **average cluster B** |
| --- | --- | --- | --- | --- | --- | --- | --- | --- | --- | --- |
| **EcoNrdR** | | | | | | | | | | |
| **AMPPNP-dATP tetramer** | 131.9 | 39.8 | 107.8 | 45.5 | 107.8 | 41.3 | 108.8 | 41.9 | 114.1 ± 10.3 | 42.1 ± 2.6 |
| **ATP-dATP tetramer** | 128.6 | 49.2 | 151.2 | 40.0 |  |  |  |  | 139.9 ± 11.3 | 44.6 ± 4.6 |
| **ATP-dATP-DNA** | 91.1 | 63.3 | 92.3 | 64.3 |  |  |  |  | 92.0 ± 0.3 | 63.8 ± 0.5 |
| **ADP-dATP tetramer^*^** | 109.8 | 34.9 |  |  |  |  |  |  | 109.8 | 34.9 |
| **ATP filament** | 136.6 | 35.4 | 137.6 | 34.2 | 138.3 | 35.2 | 139.9 | 36.3 | 138.1 ± 1.2 | 35.3 ± 0.9 |
|  |  |  |  |  |  |  |  |  |  |  |
| **ScoNrdR** | | | | | | | | | | |
| **ATP-dATP octamer** | 88.0 | 75.1 |  |  |  |  |  |  | 88.0 | 75.1 |
| **ATP-dATP-DNA** | 85.9 | 81.8 | 86.0 | 77.2 |  |  |  |  | 86.0 ± 0.1 | 79.5 ± 2.3 |
| **ATP dodecamer** | 131.1 | 68.8 |  |  |  |  |  |  | 131.1 | 68.8 |

* Chain B forms the compact tetramer in the ADP-dATP complex, chain A the infinite filaments.


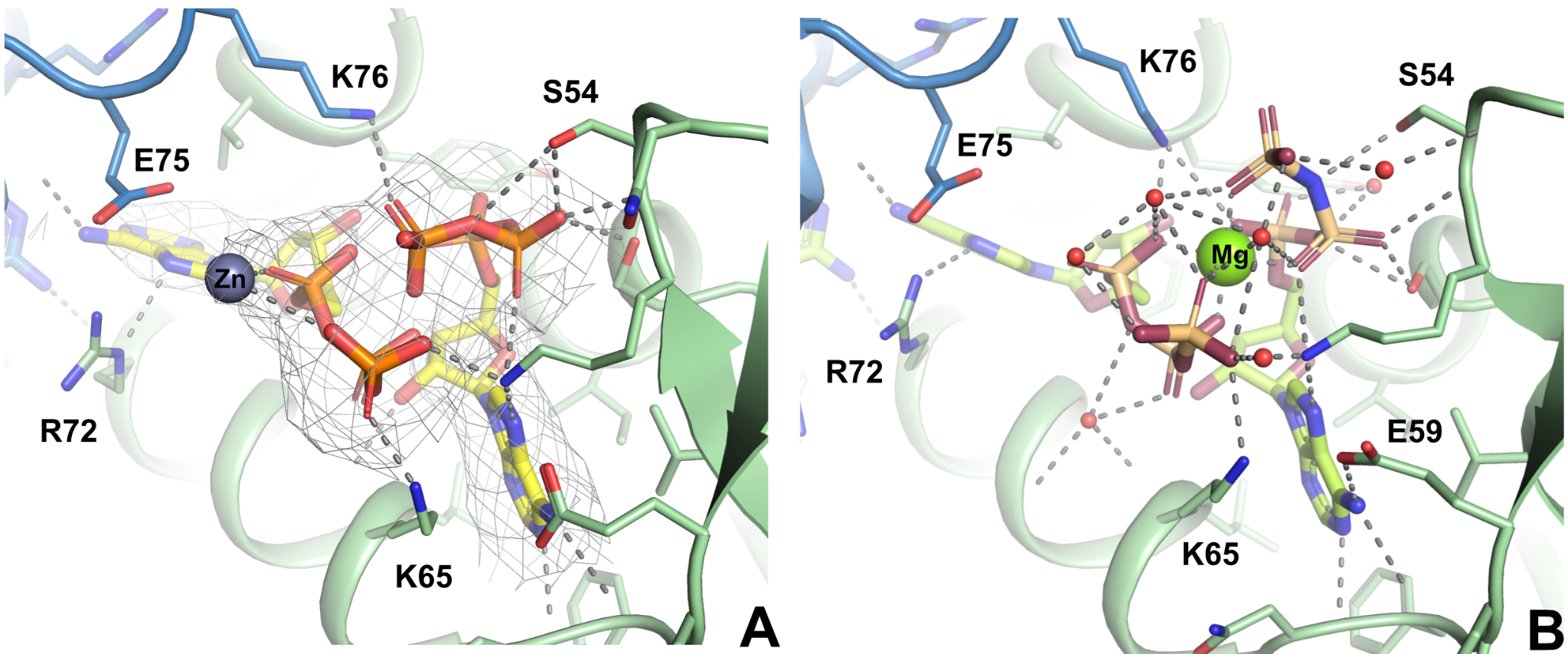


**Supporting Figure S9. Binding of nucleotides in the SeMet-EcoNrdR-dATP-ATP complex.** A) 2m|Fo|-D|Fc| electron density map and binding of ATP and dATP, with focus on the triphosphate tails and metal coordination; B) Binding of AMPPNP and dATP in the EcoNrdR AMPPNP-dATP complex for comparison. Note the lack of a Mg^2+^ ion between the triphosphate tails in the ATP-dATP complex and the presence of a Zn^2+^ ion on the outside of ATP instead. Furthermore, the g-phosphate group of dATP is bent towards ATP.

**
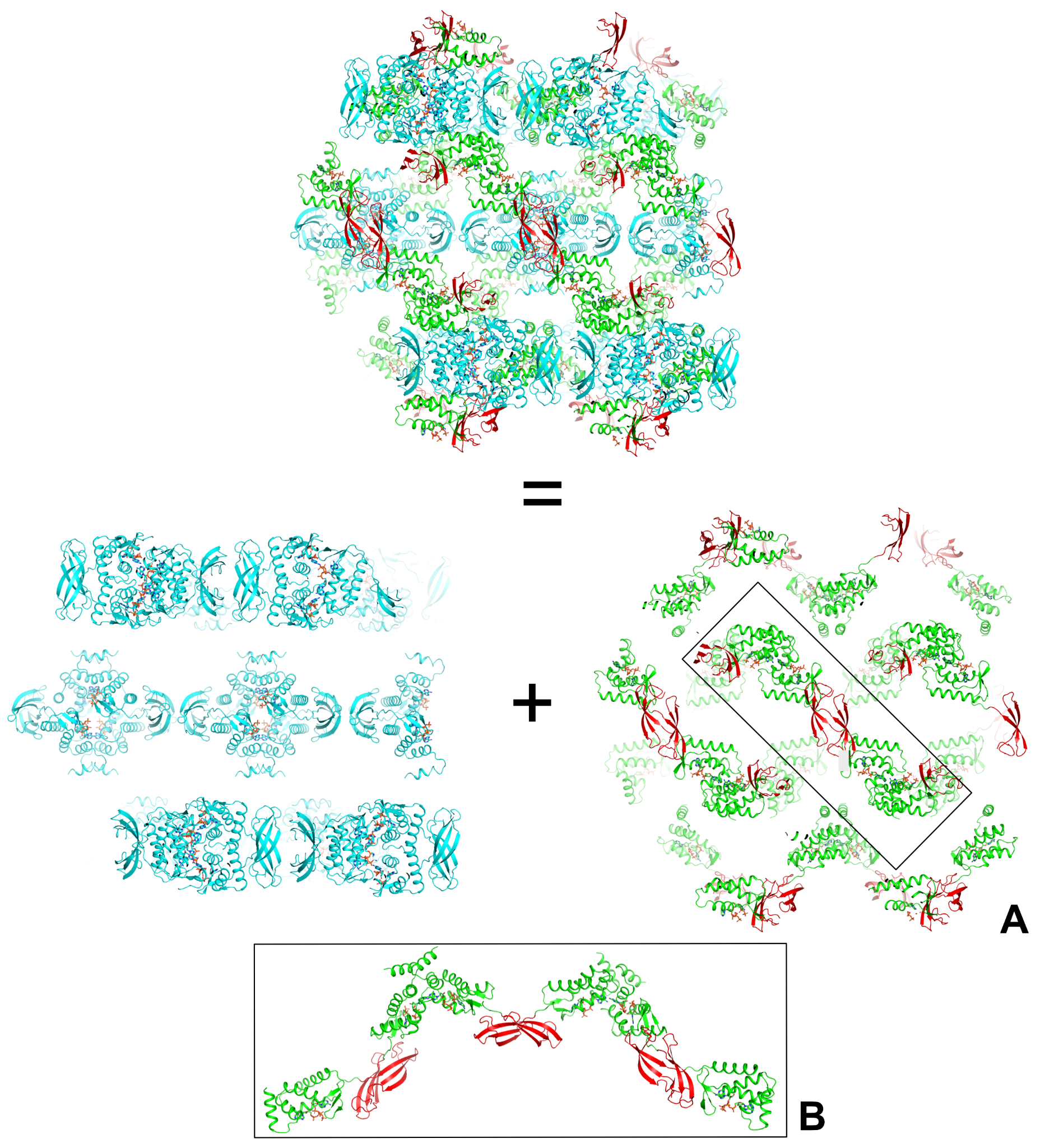
**

**Supporting Figure S10. Crystal packing in the ADP/dATP-bound form of EcoNrdR.** A) The crystal packing can be decomposed into compact tetramers (blue) decorated with infinite chains of monomers linked by alternating interactions of pairs of ATP-cone domains (green) and Zn-ribbon domains (red). B) Zoom in on part of the infinite chain.

**
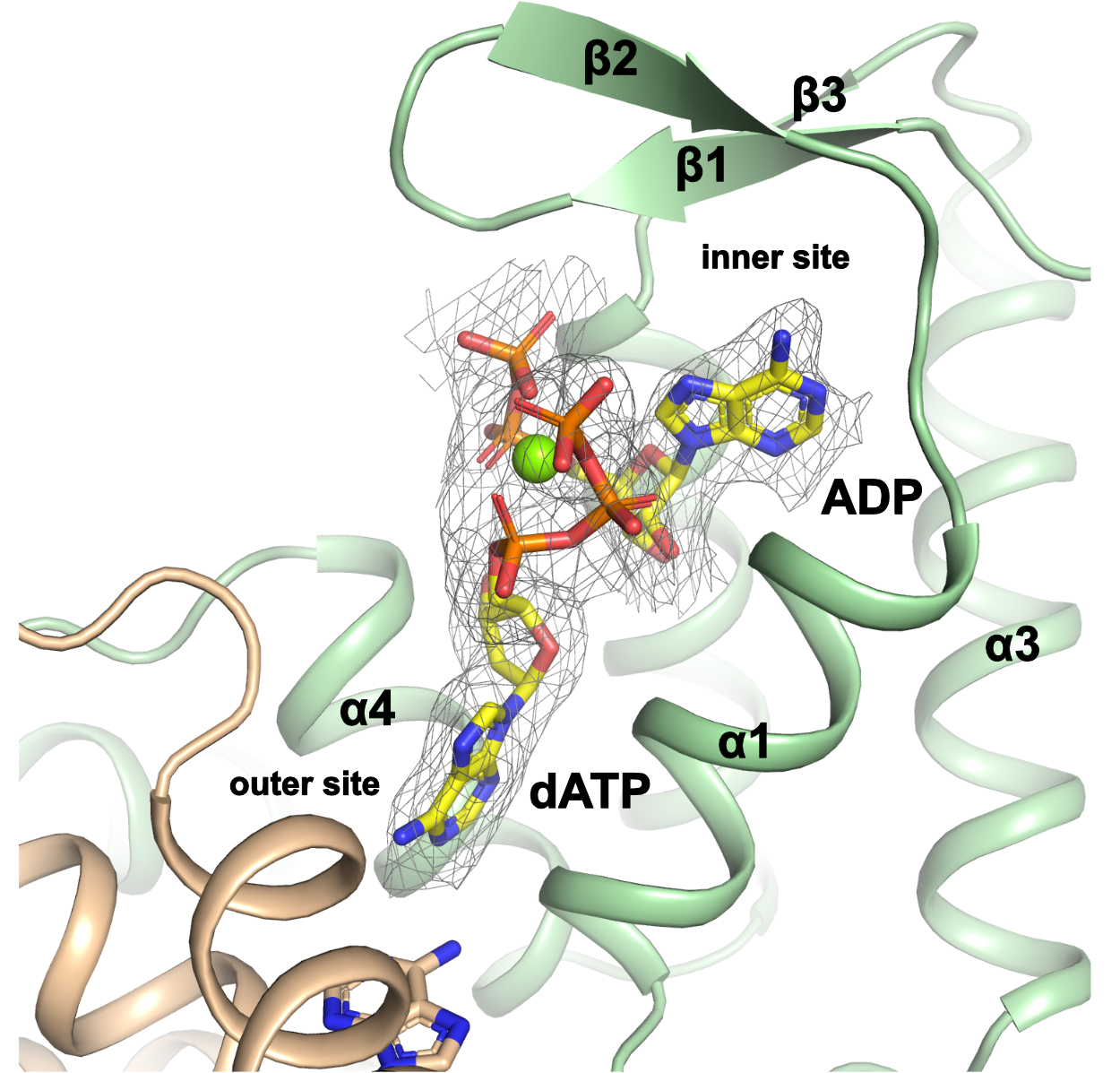
**

**Supporting Figure S11. Nucleotides in the ADP-dATP structure.** Overall view of one ATP-cone (green) showing the inner and outer sites binding ADP and dATP respectively. The second ATP-cone in the dimer is shown in beige. The Mg^2+^ ion is drawn as a green sphere. 2m|Fo|-D|Fc| electron density for the nucleotides contoured at 1.1 σ is shown as a grey mesh. Compare with binding of AMPPNP-dATP in Figure 3C,


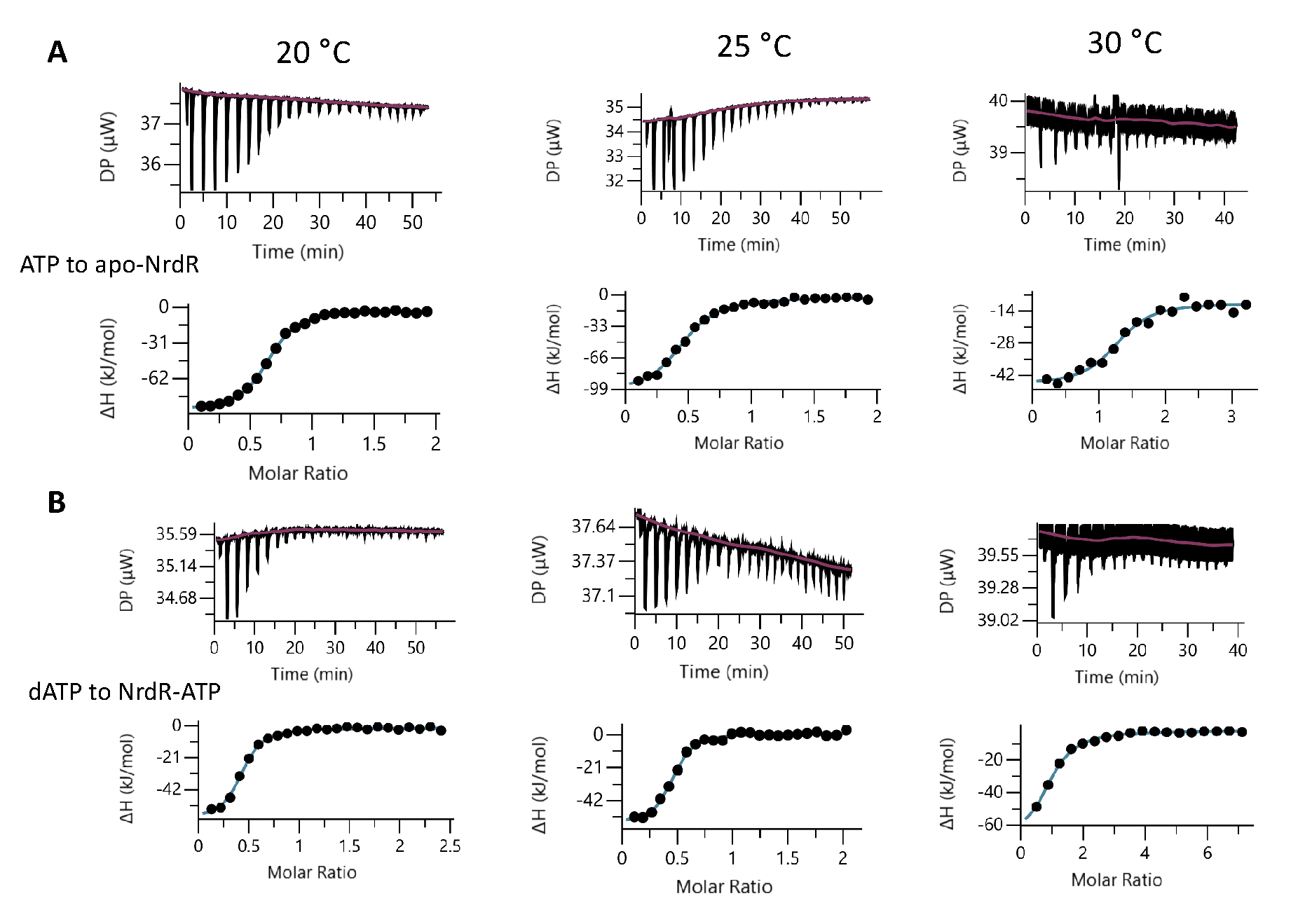


**Supporting Figure S12. ITC analyses of ligand binding to *E. coli* NrdR at 20 °C, 25 °C and 30 °C.** Representative ITC thermograms obtained by titration of ATP to apo- NrdR (A) and of dATP to NrdR loaded with ATP (B). Isothermal calorimetric enthalpy changes (upper panels) and resulting binding isotherms (lower panels) are shown. Thermodynamic parameters are presented in Table S4.

**Supporting Table S4. Thermodynamic parameters of ligand binding to NrdR at 20 °C, 25 °C and 30 °C** ^a^



^a^ Binding isotherms were fitted using a one-set-of-sites binding model. Values for ATP titration of apo-NrdR at 20 °C reported as the mean ±SD of three titrations. Other titrations are based on a single binding experiment for each ligand at each temperature. All titrations were performed as described in Materials and Methods.

**A**
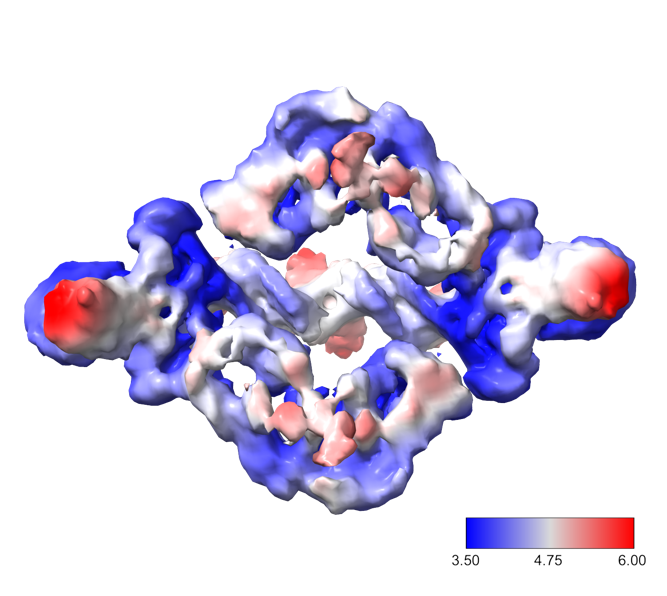
 **B**
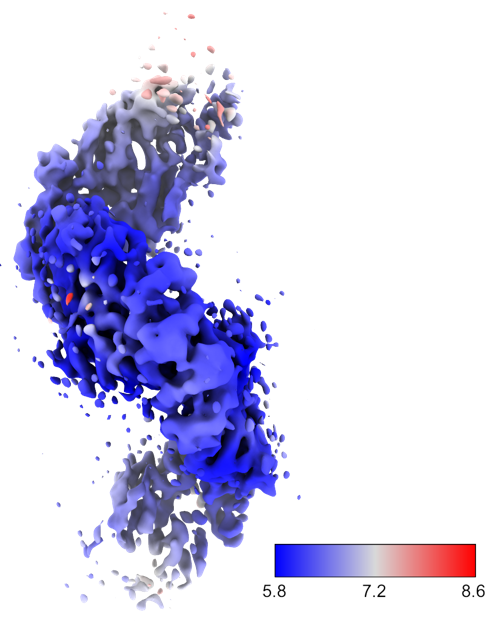


**Supporting Figure S13. Local resolution maps for the cryo-EM structures.** The globally sharpened maps from cryoSPARC (i.e. before post-processing in DeepEMhancer) are shown. Local resolution was calculated using Phenix and the two corresponding half-maps.

1. Local resolution map for the EcoNrdR-dATP-ATP-DNA complex.
2. Local resolution map for the EcoNrdR-ATP filament reconstruction.


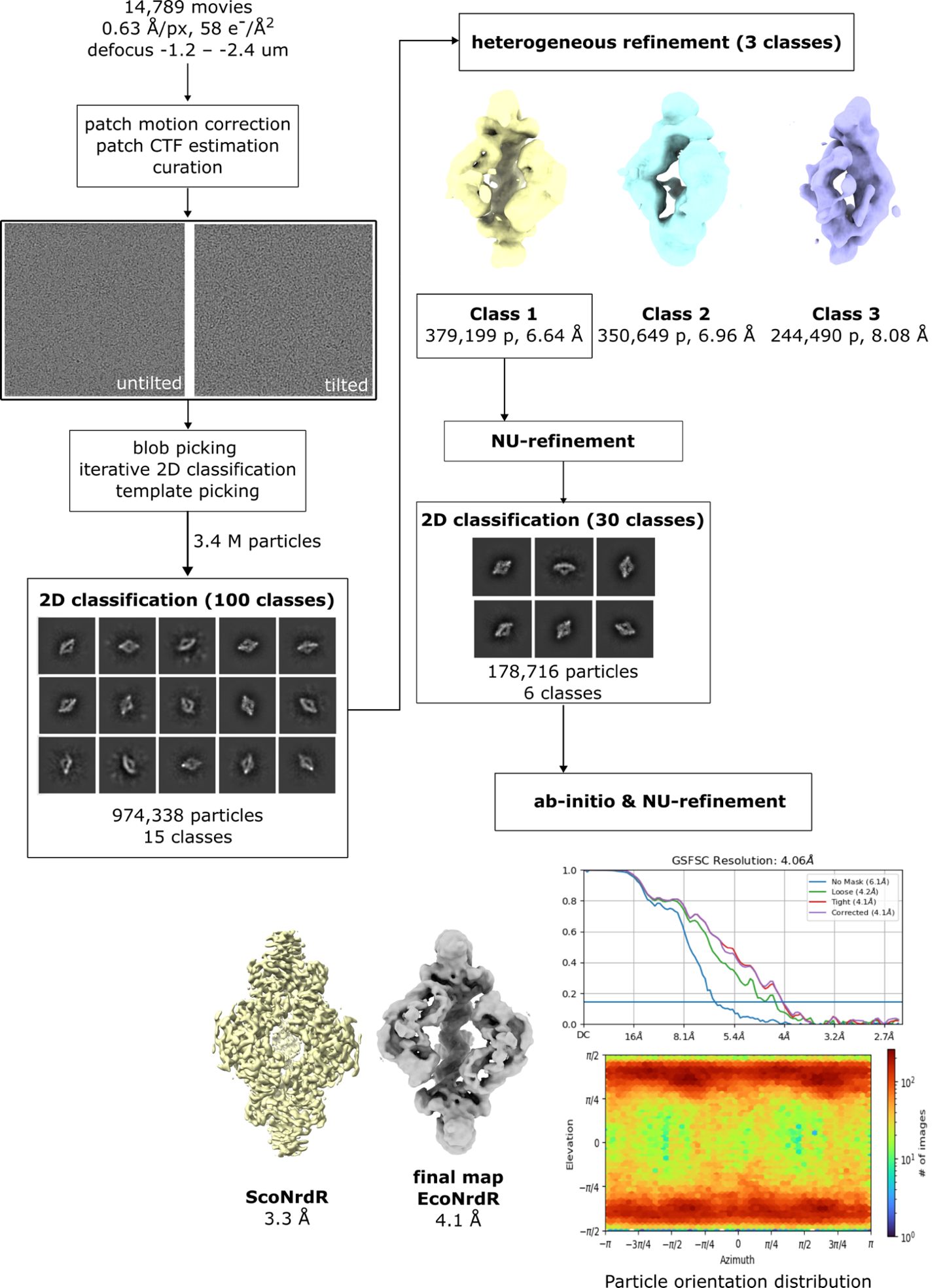


**Supporting Figure S14. Cryo-EM data processing workflow for the structure of the EcNrdR-ATP-dATP-DNA complex.** The map for the ScoNrdR complex (1) is shown in yellow for comparison.


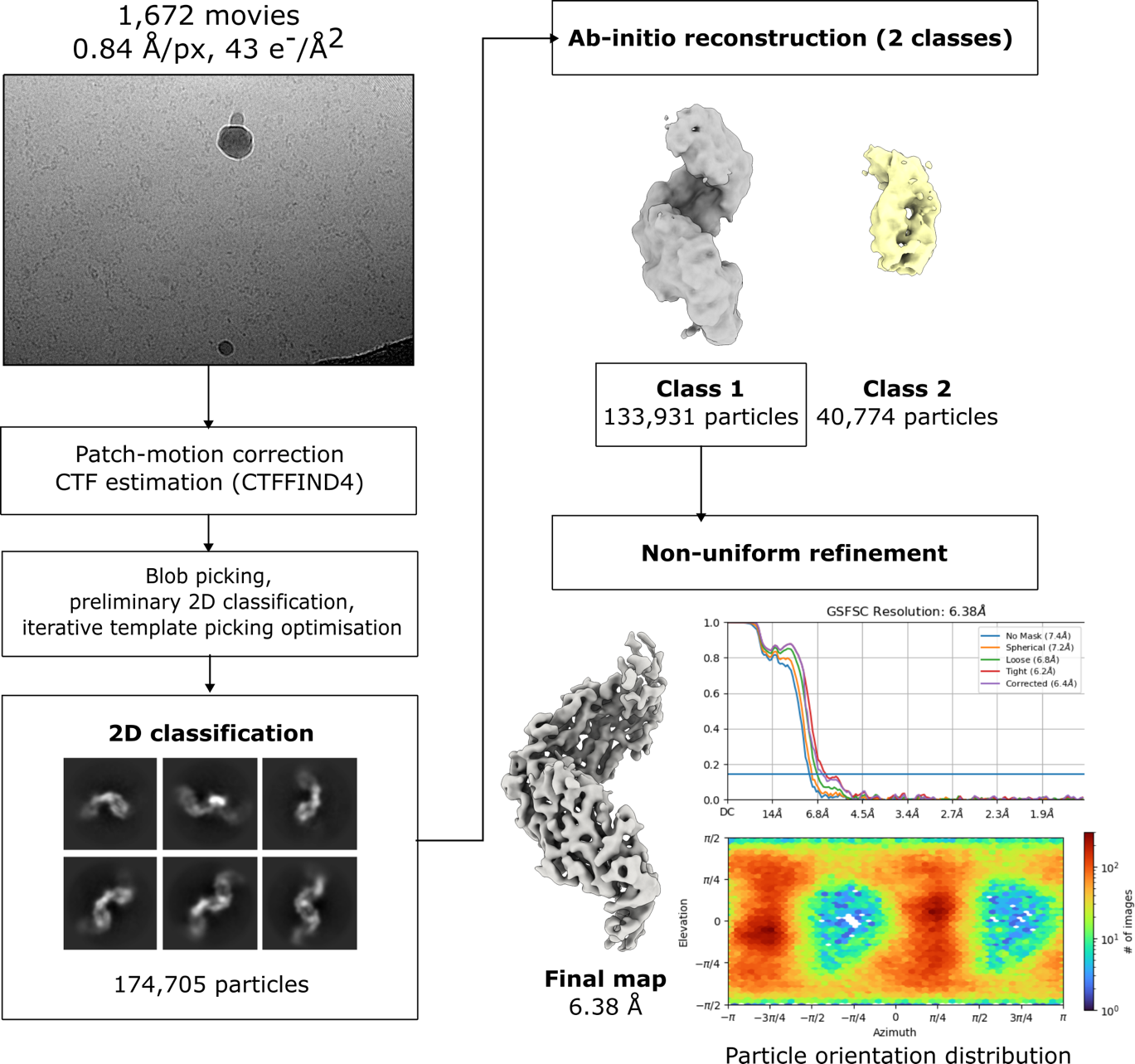


**Supporting Figure S15. Data acquisition parameters and data processing workflow for the ATP-bound NrdR filament cryo-EM dataset.**


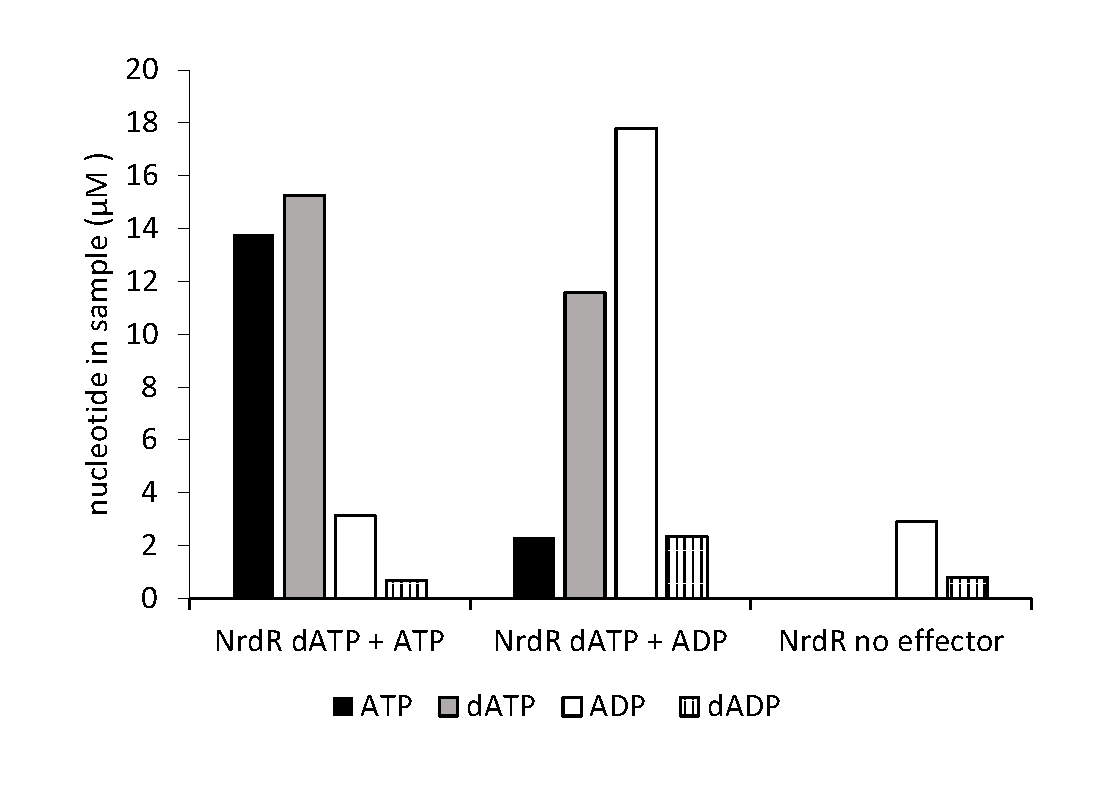


**Supporting Figure S16. Retained nucleotides in *E. coli* NrdR (35 µM) after addition of effectors and desalting.** 100 µM of as purified-NrdR in Tris-HCl 50 mM pH 8.5 at 4 C, 300 mM NaCl, 10 mM MgCl_2_, 0.5 mM TCEP were supplemented with either ATP and dATP or ADP and dATP (1 mM each), incubated at room temperature for 5 min and transferred to ice for an additional 20 min. A sample without addition of effectors was used as a control. The proteins were desalted from residual nucleotides by applying 100 µl of each sample to a NAP5 column (Cytiva) equilibrated with the same buffer, but without nucleotides. Fractions containing NrdR were collected, and its concentration was determined using Bradford. The samples containing desalted NrdR proteins were boiled for 10 minutes to release the nucleotides bound to it and centrifuged for 10 minutes at 17000 g on a table-top centrifuge. The supernatant was loaded on HPLC (Agilent) using an Agilent ZORBAX RR StableBond (C18, 4.6 x 150 mm, 3.5 µm pore size) equilibrated with buffer A (10% methanol, 50 mM potassium phosphate buffer, pH 7, 10 mM tetrabutylammonium hydroxide). A sample of 10 μl was injected and eluted at 1 ml/min with a gradient of 40%-100% buffer B (30% methanol, 50 mM potassium phosphate buffer, pH 7, 10 mM tetrabutylammonium hydroxide). Compound identification and product quantification based on peak area were performed by external calibration using injected ATP, dATP, ADP, and dADP standards. The correction for phosphate hydrolysis during boiling (10-15% determined experimentally) was taken into account in calculations.

**List of Supporting movies:**

**Supporting movie S1. Conformational differences between the EcoNrdR and ScoNrdR complexes with dATP-ATP-DNA** (1). The morph and movie were created using ChimeraX v1.7.1 (2). The movie starts with the ScoNrdD structure, morphs to the EcoNrdR structure and back again.

**Supporting movie S2. Conformational changes in EcoNrdR upon DNA binding, starting from the conformation in the dATP-AMPPNP tetramer.** The movie shows the adjustment necessary for EcoNrdR to attain a conformation where it can bind two NrdR boxes simultaneously, assuming a hypothetical starting state in which one pair of Zn ribbons is bound to one NrdR box. The DNA is in its final, highly bent form and is included as reference.

**Supporting movie S3. Conformational changes in EcoNrdR upon DNA binding, starting from the conformation in the ADP-dATP tetramer.** The movie shows the adjustment necessary for EcoNrdR to attain a conformation where it can bind two NrdR boxes simultaneously, assuming a hypothetical starting state in which only one pair of Zn ribbons is bound to one NrdR box. The DNA is in its final, highly bent form and is included as reference.

**Supporting movie S4. Conformational changes between the EcoNrdR-AMPPNP-dATP tetramer and the tetramer observed in the EcoNrdR-ATP filament.** The movie starts with the AMPPNP-bound state and ends with the ATP-bound state. The ATP-cones rotate relative to each other while the angle between ATP-cone and Zn-ribbons remains similar.

**Supporting Movie S5. Hypothetical conformational change between a state where one Zn-ribbon pair of EcoNrdR in the dATP-ATP-bound state is bound to one NrdR box in undistorted B-DNA and the final state with highly curved DNA and both Zn-ribbon pairs bound to NrdR boxes.** The B-DNA was generated from the sequence of the coding strand of the DNA in the ScoNrdR complex using ChimeraX v1.7.1 (2).

Supporting Movies S2-S5 were made using PyMOL v2.5.8 (3). Intermediate states are interpolated and do not necessarily represent true intermediates, especially for the DNA in Supporting Movie S5.

**References**

1. Rozman Grinberg, I., Martínez-Carranza, M., Bimai, O., Nouairia, G., Shahid, S., Lundin, D., Logan, D.T., Sjöberg, B.M. and Stenmark, P. (2022) A nucleotide-sensing oligomerization mechanism that controls NrdR-dependent transcription of ribonucleotide reductases. *Nat Commun*, **13**, 2700.

2. Meng, E.C., Goddard, T.D., Pettersen, E.F., Couch, G.S., Pearson, Z.J., Morris, J.H. and Ferrin, T.E. (2023) UCSF ChimeraX: Tools for structure building and analysis. *Protein Sci*, **32**, e4792.

3. Schrodinger, LLC. The PyMOL Molecular Graphics System, Version 2.5.8.
